# Are programmed cell death 1 gene polymorphisms correlated with susceptibility to rheumatoid arthritis? A meta-analysis

**DOI:** 10.1097/MD.0000000000007805

**Published:** 2017-09-01

**Authors:** Yuming Zou, Ziteng Zhang, Yangang Liu, Denghui Liu, Weidong Xu

**Affiliations:** a Department of Orthopedics, Changhai Hospital, The First Affiliated Hospital of the Second Military Medical University; b Department of Health Toxicology, College of Tropical Medicine and Public Health, Second Military Medical University, Shanghai, China.

**Keywords:** meta-analysis, programmed cell death 1, rheumatoid arthritis

## Abstract

**Background:**

Several studies investigated the relationship between programmed cell death 1 (*PDCD1*) gene polymorphisms and rheumatoid arthritis (RA) risk, but the results were controversial. To explore whether *PDCD1* gene polymorphisms have an effect on RA risk, we conducted this meta-analysis to investigate the relationships between *PDCD1* polymorphisms (rs36084323 [PD-1.1 G/A], rs11568821 [PD-1.3 G/A] and rs2227981 [PD-1.5 C/T]) and RA risk under 4 genetic models.

**Methods:**

PubMed, EMBASE, Web of Science, Cochrane Library China National Knowledge Infrastructure (CNKI), and Chinese Biomedical Literature Database (CBLM) were systematically searched for all eligible case-control studies. The last search was updated on September 10, 2016. Studies were accessed using Newcastle-Ottawa Scale case control study (NOS), and the combined effect size was calculated using STATA software, version 12.0. The pooled odds ratio (OR) with 95% confidence interval (CI) was calculated to assess the association. Heterogeneity analysis and subgroup analysis were also performed. Sensitivity analysis and publication bias were also performed if necessary.

**Results:**

This meta-analysis included 6 studies. The result demonstrated null association between rs36084323 (PD-1.1 G/A) polymorphism and RA susceptibility in all 4 genetic models. With regard to rs11568821 (PD-1.3 G/A), statistically significant association with RA risk was observed under allele model in Caucasians (allele model A vs G, OR = 1.19, 95% CI = 1.03–1.41). There was no significant association between rs2227981 (PD-1.5 C/T) polymorphism and RA risk.

**Conclusion:**

The present study suggests that mutant A allele in rs11568821 (PD-1.3 G/A) might increase the susceptibility to RA in Caucasians.

## 1 Introduction

Rheumatoid arthritis (RA) is a chronic inflammatory, autoimmune disease characterized by joint inflammation and synovitis, which could lead to cartilage and bone destruction, disability, and eventually, to systemic complications including cardiovascular and pulmonary disorders.^[1]^ Interactions between genetic susceptibility and environmental factors have been found to play an important role in the pathogenesis and progression of RA.^[2]^ Genetic risks for RA have been acknowledged for years, various new genetic risk factors for RA were identified with the help of genome-wide association studies (GWAS), such as HLA-DRB1, PADI4, PTPN22, TNFAIP3, STAT4, AFF3, and CCR6.^[3–6]^ Despite these risk factors, the contribution of these individual risk loci to the development of RA appears to be variable,^[7]^ the exact pathogenesis of RA still needs to be elucidated.

Programmed cell death 1 (*PDCD1* or PD1), whose gene is localized on chromosome 2q37.3, is a member of the CD28/B7 family receptors, encoding a 55 kDa immunosuppressive receptor in T cells, B cells, and myeloid cells.^[8]^*PDCD1* is a necessary negative regulator of autoimmunity regulation; the interactions between *PDCD1* and its ligands, PD-L1and PD-L2, have a negative effect in T cell receptor-mediated lymphocyte proliferation and cytokine secretion, and could inhibit CD28-mediated costimulation.^[9]^ It also regulated the induction and maintenance of T cell tolerance, which could protect tissues from effector T cell responses-mediated tissue damage.^[8,10,11]^ For instance, a recent study indicated that *PDCD1* upregulated on regulatory T cells enhances the suppression of CD8+ T cell immune response via the interaction with PD-L1 expressed on CD8+ T cells,^[12]^ and another study showed that genetic absence of *PDCD1* promoted accumulation of terminally differentiated CD8+ T cells.^[13]^ The lack of *PDCD1* could result in the development of autoimmune diseases, such as systemic lupus erythematosus (SLE),^[14]^ type I diabetes mellitus (T1DM),^[15]^ and ankylosing spondylitis (AS).^[13]^

RA is a chronic autoimmune and inflammatory disorder, featured by immune-mediated inflammatory circumstances of the synovium. In RA synovial fluid, the PD-1^+^ T cells are enriched and consist of a unique allergic T cell subset in synovial fluid of RA patients.^[16]^ The *PDCD1* gene has several polymorphisms, including rs36084323 (PD-1.1 G/A), rs11568821 (PD-1.3 G/A), rs2227981 (PD-1.5 C/T), and rs2227982 (PD-1.9 C/T), which could influence the biological function of *PDCD1*. *PDCD1* gene polymorphisms were found to be correlated with the risk of several autoimmune diseases, such as SLE, RA, diabetes mellitus, and multiple sclerosis (MS).^[17]^ Several studies aimed to investigate the relationship between *PDCD1* gene polymorphisms and RA risk, but the results were controversial.^[18–23]^ For instance, Prokunina et al confirmed there is an association between rs11568821 (PD-1.3 G/A) polymorphism and the risk of RA in the Caucasian population, which was supported by the study of Tahoori et al.^[20]^ However, Iwamoto et al^[21]^ and Liu et al^[19]^ hold a different opinion. In order to provide a comprehensive assessment of the association between the polymorphisms of *PDCD1* and RA risk, we conducted this meta-analysis.

## 2 Material and methods

No ethical approval and informed consent are necessary for this meta-analysis, and it was reported following the PRISMA (Preferred Reporting Items for Systematic Reviews and Meta-Analyses) statement.^[24]^

### 2.1 Literature research

We systematically searched databases including PubMed, EMBASE, Web of Science, Cochrane Library China National Knowledge Infrastructure (CNKI), and Chinese Biomedical Literature Database (CBLM) for all eligible case-control studies with a retrieval strategy. The last search was updated on September 10, 2016. The following search terms were used: rheumatoid arthritis OR RA and programmed cell death 1 OR *PDCD1* OR PD-1 OR PD-1.1 OR rs36084323 OR PD-1.3 OR rs11568821 OR PD-1.5 OR rs2227981 OR PD-1.9 OR rs2227982, and polymorphism OR polymorphisms OR single nucleotide polymorphism OR SNP OR variation. Publication language was not restricted in our study selection. Additional eligible studies were identified by searching the references of all the selected articles.

### 2.2 Inclusion criteria and publication quality assessment

Inclusion criteria of this meta-analysis were as follows: case-control studies; investigation of the relationship between RA risk with at least 1 of *PDCD1* gene polymorphisms, including rs36084323 (PD-1.1 G/A), rs11568821 (PD-1.3 G/A), rs2227981 (PD-1.5 C/T), and rs2227982 (PD-1.9 C/T); and enough data were provided for analysis. Newcastle-Ottawa Scale was used to assess the quality of all the eligible studies in the following 3 aspects: selection, comparability, and exposure. Scores ranging from 0 to 9 were used to evaluate the quality of the original studies. A score of 6 or above was considered as high quality.

### 2.3 Data extraction

All the eligible studies were independently reviewed by 2 authors. Any disagreement about data was settled through a team discussion until a consensus was reached. For each study, the following data were collected: author list, year of publication, ethnicity, genotyping method, genotypes frequency in each polymorphism, diagnostic criteria, matching criteria, source of RA, source of control, and NOS scores.

### 2.4 Statistical analysis

We performed a meta-analysis to assess the association between *PDCD1* polymorphisms and RA susceptibility. Chi-square test was used to evaluate whether the genotype distributions of each control group followed the Hardy-Weinberg equilibrium (HWE), significant *P* < .05 was considered statistically significant. Odds ratio (OR) and 95% confidence interval (CI) were calculated to evaluate the association between *PDCD1* polymorphisms and RA risk in 4 genetic models which were dominant, recessive, codominant, and additive. Subgroup analysis was performed based on ethnicity (Asians or Caucasians). The chi-squared-based Q statistical test and *I*^2^ metric value were used to assess the statistical heterogeneity between studies. If the *I*^2^ value is >50% or *P*_*Q*_ < .10, a difference in statistical heterogeneity existed, ORs were pooled by the random-effect model, otherwise, the fixed-effect model was applied.^[25]^ Publication bias was examined by a funnel plot and Egger test. If the *P* value in Egger test was less than .05, publication bias was statistically significant. Otherwise, the study was considered to have no publication bias.

All statistical analyses were performed using Stata 12.0 software (StataCorp, College Station, TX) and all *P* values were considered 2-sided, unless otherwise stated.

## 3 Results

### 3.1 Study characteristics

**Figure 1:**
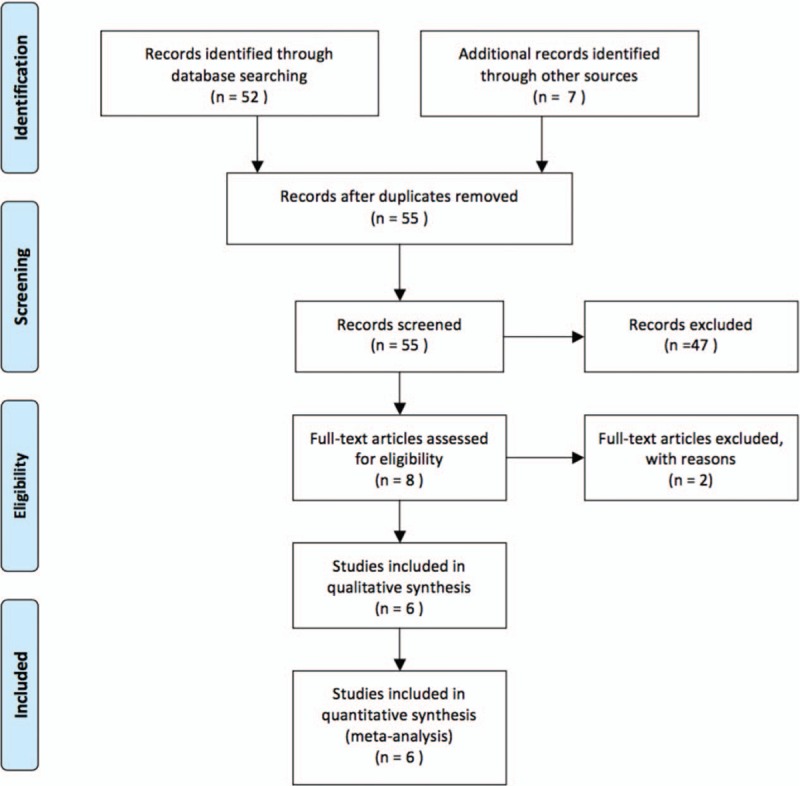
The study selection and inclusion process.

A total of 6 studies^[18–23]^ investigated the relationship of *PDCD1* polymorphisms and RA risk fulfilled the eligibility criteria. The study selection process is shown in Figure 1. There were 4 studies involving 2094 cases and 1591 controls that reported the relationship between rs36084323 (PD-1.1 G/A) polymorphism and RA.^[19–22]^ Three studies involving 1382 cases and 3720 controls focused on the relationship between rs11568821 (PD-1.3 G/A) polymorphism and RA.^[18–21,23]^ Another 3 studies including 1985 RA cases and 1400 controls studied the relationship between rs2227981 (PD-1.5 C/T) polymorphism and RA.^[19,21,22]^ Moreover, rs11568821 (PD-1.3 G/A) was nonpolymorphic in Asians and so was rs2227981 (PD-1.5 C/T) in Caucasians. Because there were only 2 studies exploring rs2227982 (PD-1.9 C/T) polymorphism, we excluded it in our study. The general demographics characters of all these studies are shown in Table 1. The genotype distribution in the control subjects in all studies was consistent with HWE.

**Table 1:**
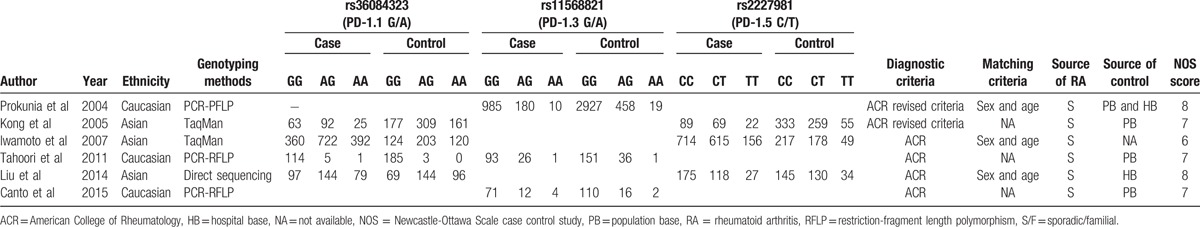
General characteristics of the eligible studies.

### 3.2 Meta-analysis results

#### 3.2.1 rs36084323 (PD-1.1 G/A) polymorphism and RA susceptibility in Caucasians or in Asians

**Figure 2:**
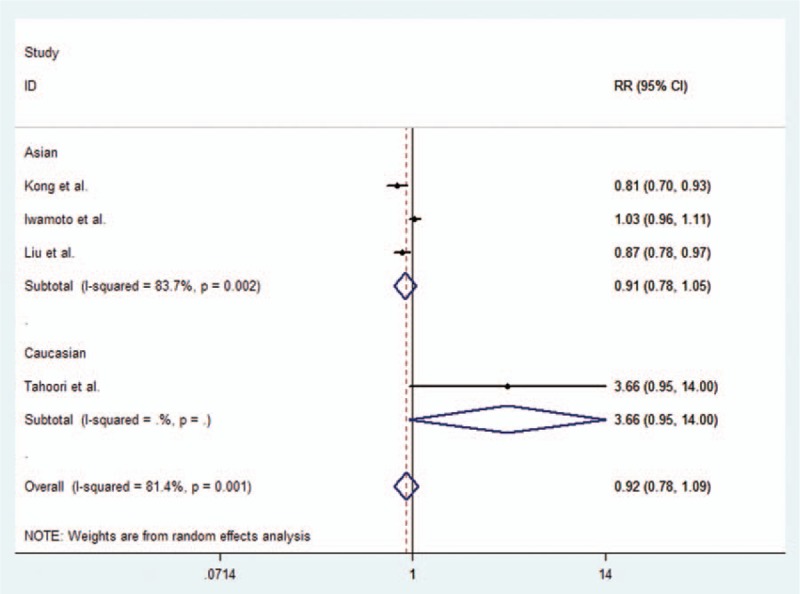
Forest plot describing the association between rs36084323 (PD-1.1 G/A) polymorphism and RA risk under the allele contrast model (A vs G).

For the association between rs36084323 (PD-1.1 G/A) and the risk of RA, 4 studies were included according to the match criteria. Significant heterogeneity existed in all genetic models, so we applied random-effect model. We found null association between rs36084323 (PD-1.1 G/A) and RA risk in any of the 4 genetic models based on random-effect model. Because there was only 1 study in Caucasian population, we conduct ethnicity subgroup analysis in Asians. Similarly, we failed to find any association in any of the 4 genetic models based on random-effect model. All the results are shown in Table 2. The forest plot for the additive model based on the random-effect model was selected to perform the pooled OR as shown in Figure 2.

**Table 2:**
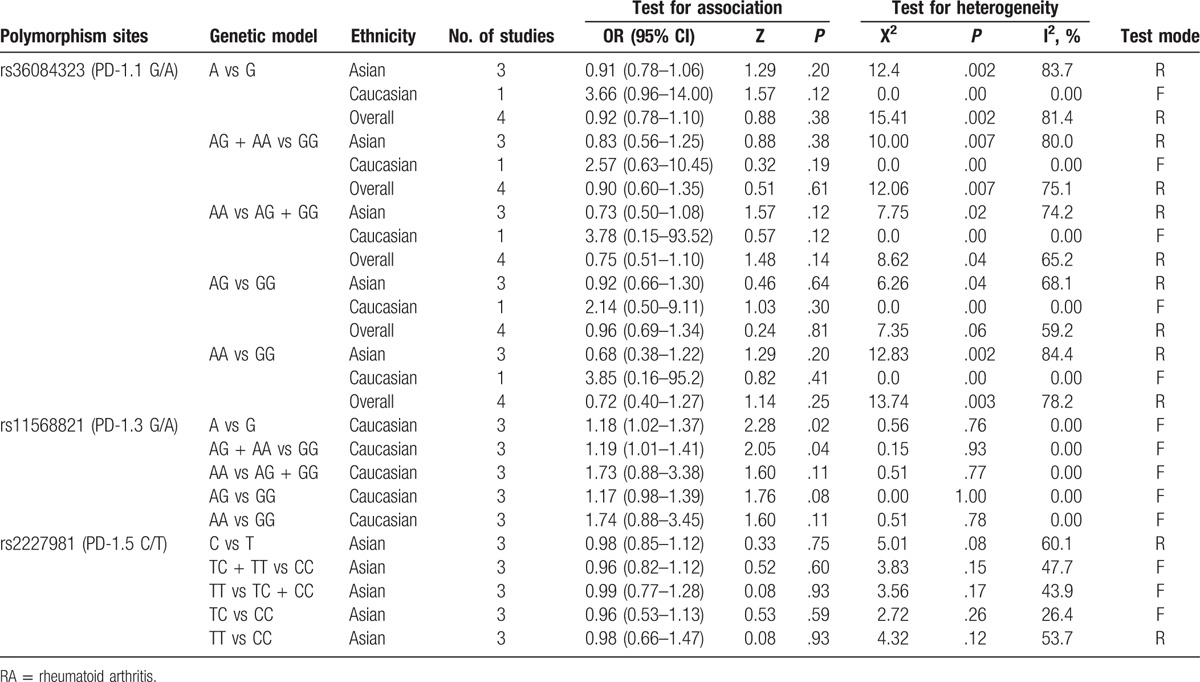
Results of genetic models for rs36084323 (PD-1.1 G/A), rs11568821 (PD-1.3 G/A), and rs2227981 (PD-1.5 C/T) polymorphisms and RA.

#### 3.2.2 rs11568821 (PD-1.3 G/A) polymorphism and RA susceptibility in Caucasians or in Asians

**Figure 3:**
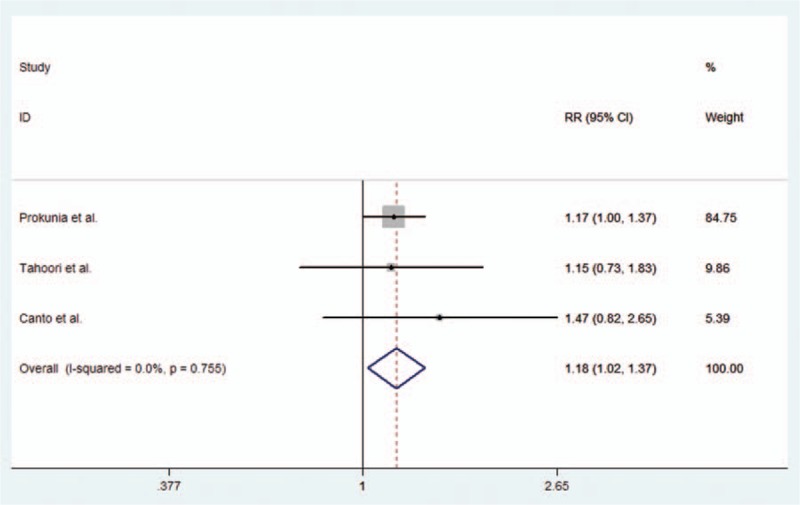
Forest plot describing the association between rs11568821 (PD-1.3 G/A) polymorphism and RA risk under the allele contrast model (A vs G).

As for rs11568821 (PD 1.3 G/A), 3 studies were included. We applied fixed-effect model because there was no significant heterogeneity in any of the 4 genetic models. There was no rs11568821 (PD-1.3 G/A) polymorphism in Asian populations, we only conducted analysis in Caucasian. We observed significant correlation between rs11568821 (PD-1.3 G/A) and RA risk in addictive model and dominant model (A vs G: OR = 1.20, 95% CI = 1.03–1.41; AG + AA vs GG: OR = 1.19, 95% CI = 1.01–1.41) in Caucasian. Table 2 showed the results of statistical analyses. The forest plot for the additive model is given in Figure 3.

#### 3.2.3 rs2227981 (PD-1.5 C/T) polymorphism and RA susceptibility in Asians

**Figure 4:**
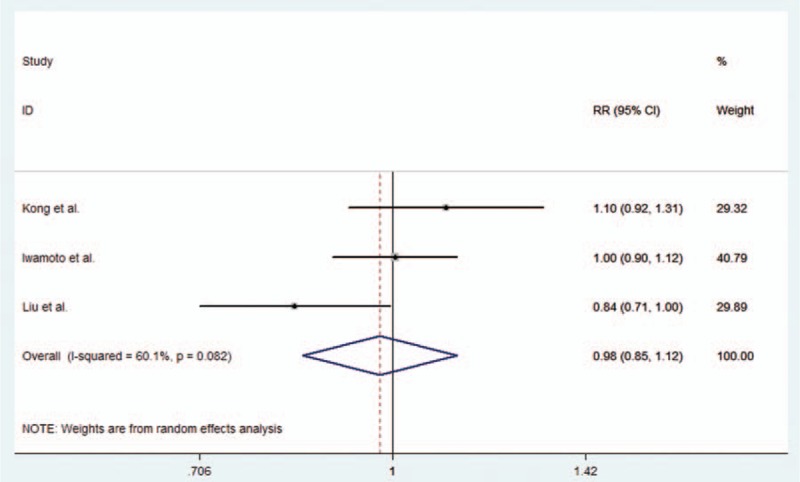
Forest plot describing the association between rs2227981 (PD-1.5 C/T) polymorphism and RA risk under the allele contrast model (T vs C).

With regard to rs2227981 (PD-1.5 C/T), our study included 3 studies, which were conducted in Asian population. The results indicated that there was no significant association between rs2227981 (PD-1.5 C/T) polymorphisms and RA risk in any of 4 genetic models. All the results were listed in Table 2. The forest plot for the additive model is given in Figure 4.

#### 3.2.4 Publication bias

Publication bias was assessed by Begg funnel plots and Egger test. Significant publication bias was not observed in any meta-analysis of the 3 polymorphisms under addictive model (*t* = 0.18, *P* = .87 for rs36084323 [PD-1.1 G/A]; *t* = 0.97, *P* = .51 for rs11568821 [PD-1.3 G/A]; *t* = −0.18, *P* = .89 for rs2227981 [PD-1.5 C/T]). The shape of the Begg funnel plots in the overall population did not reveal any obvious asymmetry.

## 4 Discussion

RA is one of the most common and the typical representative of autoimmune diseases, characterized by synovial tissue inflammation and progressive destruction cartilage, which is one of the strongest predictors of long-term outcome and disability in RA patients.^[26,27]^ Environmental and genetic factors participate in the pathogenesis of RA.^[28,29]^ Recently, studies have focused on the identification of genes that influenced the susceptibility to this disease, among them was *PDCD1*, which was an important negative regulator of autoimmunity. This gene not only plays an important role in the regulation of the induction and maintenance of T cell tolerance, but also inhibits effector T cell responses and protects tissues from autoimmune-mediated tissue damage.^[8,10,11]^ Therefore, it is considered to be a strong candidate gene responsible for autoimmune diseases, especially for RA.^[22]^ As indicated, *PDCD1* gene polymorphisms are associated with autoimmune disorders, such as SLE,^[29]^ T1D,^[30]^ and AS^[31]^ (one of our previous studies).

Though a number of studies have investigated the association between *PDCD1* polymorphism and susceptibility to RA, the results remain controversial. In this meta-analysis, we quantitatively evaluated the correlation between 3 SNPs of *PDCD1* gene and RA susceptibility.

The rs36084323 (PD-1.1 G/A) is located in the promoter of *PDCD1* gene (Gene Bank accession no. AF363458). Transcription initiation is an important part of regulation in gene expression. Polymorphisms that occurred in the promoter could affect the normal process of gene activation and transcriptional initiation, thus increase or decrease the quantity of mRNA and protein.^[32]^ Theoretically, PD-1.1 should be the most likely polymorphism site associated with RA risk. However, we failed to detect significant association of rs36084323 (PD-1.1 G/A) polymorphism and RA risk according to our results. When further stratified by ethnicity, our results indicated null association in Asian populations. The limited studies and generally small sample size may affect the accuracy of this result especially given that only 1 study was conducted in Caucasian populations.

For rs11568821 (PD-1.3 G/A) polymorphism, statistical significance was observed between rs11568821 (PD-1.3 G/A) polymorphism and risk of RA. This result was consistent with a previous meta-analysis by Lee et al.^[30]^ Considering only 2 studies were involved in their study, our result improved the credibility of the conclusion. Even so, extra cautious should be taken because of the generally limited number of studies and the OR value, which was close to 1. The rs11568821 (PD-1.3 G/A) is located in intron 4, in general, polymorphic sequence variations are considered to be rather harmless, especially if located in noncoding parts of a gene. It may be that the Rs11568821 (PD-1.3 G/A) is not itself the responsible allele, but is in linkage disequilibrium with the true causative allele. Although rs11568821 (PD 1.3 G/A) polymorphism has been found to be significantly associated with the risk of developing RA, the interactive function of *PDCD1* SNPs and other genes may have an important role in the etiology and development of RA.^[22]^

Similar to rs36084323 (PD-1.1 G/A), there was null significant association between rs2227981 (PD-1.5 C/T) and susceptibility to RA under 4 genetic models. All 3 studies involved in the relationship between rs2227981 (PD-1.5 C/T) polymorphism and RA risk were from Asian populations. In order to further verify this result, studies from other populations should be addressed.

Some limitations of this study should be addressed. First, the number of studies fulfilled the eligibility criteria and finally included in this study were limited. Among which 4 studies reported the polymorphism of rs36084323 (PD-1.1 G/A) (3 from Asians and 1 from Caucasians); 5 studies reported the polymorphism of rs11568821 (PD-1.3 G/A) (2 from Asians and 3 from Caucasians); 3 studies reported the polymorphism of rs2227981 (PD-1.5 C/T) (all from Asians). This could not provide sufficient statistical power to study the exact effect of SNPs in *PDCD1* gene, especially in different ethnic groups, so our analysis may have been underpowered. Second, confounding factors, publication bias, and heterogeneity may have distorted the results. Furthermore, we primarily collected data about the rs2227982 (PD-1.9 C/T) polymorphism and the RA risk, but only 2 studies were eligible and they were from different ethnicity so thus it were not included in our study.

In conclusion, we conducted a meta-analysis of all the eligible studies related to the rs36084323 (PD-1.1 G/A), rs11568821 (PD-1.3 G/A), and rs2227981 (PD-1.5 C/T), and susceptibility to RA. The results show that no significant relationships were observed between rs36084323 (PD-1.1 G/A) and rs2227981 (PD-1.5 C/T) polymorphisms and RA risk, neither in Caucasians nor Asians, while rs11568821 (PD-1.3 G/A) was observed to be significantly associated with risk of RA in Caucasians (OR = 1.18, 95% CI = 1.02–1.37). The mutant A allele in rs11568821 (PD-1.3 G/A) polymorphism represents a higher risk of RA in Caucasians. However, as a limited number of eligible studies were included in this work, the reported results should be cautiously interpreted. For the purpose of obtaining more reliable results, it is necessary to conduct better-designed studies based on larger sample sizes in the future.

## References

[1] Crowson CS Liao KP Davis JM III Rheumatoid arthritis and cardiovascular disease. Am Heart J 2013;166:622–8. 24093840 10.1016/j.ahj.2013.07.010 PMC3890244

[2] Turk SA van Beers-Tas MH van Schaardenburg D Prediction of future rheumatoid arthritis. Rheum Dis Clin North Am 2014;40:753–70. 25437290 10.1016/j.rdc.2014.07.007

[3] Stahl EA Raychaudhuri S Remmers EF Genome-wide association study meta-analysis identifies seven new rheumatoid arthritis risk loci. Nat Genet 2010;42:508–14. 20453842 10.1038/ng.582 PMC4243840

[4] Neidhart M Karouzakis E Genetics: a new interpretation of genetic studies in RA. Nat Rev Rheumatol 2014;10:199–200. 24567061 10.1038/nrrheum.2014.21

[5] Orozco G Viatte S Bowes J Novel rheumatoid arthritis susceptibility locus at 22q12 identified in an extended UK genome-wide association study. Arthritis Rheumatol 2014;66:24–30. 24449572 10.1002/art.38196 PMC4285161

[6] de Rooy DP Zhernakova A Tsonaka R A genetic variant in the region of MMP-9 is associated with serum levels and progression of joint damage in rheumatoid arthritis. Ann Rheum Dis 2014;73:1163–9. 23696630 10.1136/annrheumdis-2013-203375

[7] Gerlag DM Norris JM Tak PP Towards prevention of autoantibody-positive rheumatoid arthritis: from lifestyle modification to preventive treatment. Rheumatology 2016;55:607–14. 26374913 10.1093/rheumatology/kev347 PMC4795536

[8] Keir ME Butte MJ Freeman GJ PD-1 and its ligands in tolerance and immunity. Annu Rev Immunol 2008;26:677–704. 18173375 10.1146/annurev.immunol.26.021607.090331 PMC10637733

[9] Bennett F Luxenberg D Ling V Program death-1 engagement upon TCR activation has distinct effects on costimulation and cytokine-driven proliferation: attenuation of ICOS, IL-4, and IL-21, but not CD28, IL-7, and IL-15 responses. J Immunol 2003;170:711–8. 12517932 10.4049/jimmunol.170.2.711

[10] Dai S Jia R Zhang X The PD-1/PD-Ls pathway and autoimmune diseases. Cell Immunol 2014;290:72–9. 24908630 10.1016/j.cellimm.2014.05.006

[11] Freeman GJ Long AJ Iwai Y Engagement of the PD-1 immunoinhibitory receptor by a novel B7 family member leads to negative regulation of lymphocyte activation. J Exp Med 2000;192:1027–34. 11015443 10.1084/jem.192.7.1027 PMC2193311

[12] Park HJ Park JS Jeong YH Correction: PD-1 upregulated on regulatory T cells during chronic virus infection enhances the suppression of CD8+ T cell immune response via the interaction with PD-L1 expressed on CD8+ T cells. J Immunol 2015;195:5841–2. 26637664 10.4049/jimmunol.1502256

[13] Zhou L Zhang Y Xu H Decreased programmed death-1 expression on the T cells of patients with ankylosing spondylitis. Am J Med Sci 2015;349:488–92. 25881983 10.1097/MAJ.0000000000000468

[14] Prokunina L Castillejo-Lopez C Oberg F A regulatory polymorphism in PDCD1 is associated with susceptibility to systemic lupus erythematosus in humans. Nat Genet 2002;32:666–9. 12402038 10.1038/ng1020

[15] Wang J Yoshida T Nakaki F Establishment of NOD-Pdcd1−/− mice as an efficient animal model of type I diabetes. Proc Natl Acad Sci USA 2005;102:11823–8. 16087865 10.1073/pnas.0505497102 PMC1188011

[16] Hatachi S Iwai Y Kawano S CD4+ PD-1+ T cells accumulate as unique anergic cells in rheumatoid arthritis synovial fluid. J Rheumatol 2003;30:1410–9. 12858435

[17] Zamani MR Aslani S Salmaninejad A PD-1/PD-L and autoimmunity: a growing relationship. Cell Immunol 2016;310:27–41. 27660198 10.1016/j.cellimm.2016.09.009

[18] Canto LM Farias TD Medeiros MD Association of PDCD1 polymorphism to systemic lupus erythematosus and rheumatoid arthritis susceptibility. Rev Bras Reumatol 2015;56:483–9. 10.1016/j.rbre.2015.07.008 27914594

[19] Liu C Jiang J Gao L A promoter region polymorphism in PDCD-1 gene is associated with risk of rheumatoid arthritis in the Han Chinese population of southeastern China. Int J Genomics 2014;2014:247637. 24804191 10.1155/2014/247637 PMC3996357

[20] Tahoori MT Pourfathollah AA Akhlaghi M Association of programmed cell death-1 (PDCD-1) gene polymorphisms with rheumatoid arthritis in Iranian patients. Clin Exp Rheumatol 2011;29:763–7. 21961966

[21] Iwamoto T Ikari K Inoue E Failure to confirm association between PDCD1 polymorphisms and rheumatoid arthritis in a Japanese population. J Hum Genet 2007;52:557–60. 17468813 10.1007/s10038-007-0145-2

[22] Kong EK Prokunina-Olsson L Wong WH A new haplotype of PDCD1 is associated with rheumatoid arthritis in Hong Kong Chinese. Arthritis Rheum 2005;52:1058–62. 15818672 10.1002/art.20966

[23] Prokunina L Padyukov L Bennet A Association of the PD-1.3A allele of the PDCD1 gene in patients with rheumatoid arthritis negative for rheumatoid factor and the shared epitope. Arthritis Rheum 2004;50:1770–3. 15188352 10.1002/art.20280

[24] Moher D Liberati A Tetzlaff J Preferred reporting items for systematic reviews and meta-analyses: the PRISMA statement. BMJ 2009;339:b2535. 19622551 10.1136/bmj.b2535 PMC2714657

[25] Mantel N Haenszel W Statistical aspects of the analysis of data from retrospective studies of disease. J Natl Cancer Inst 1959;22:719–48. 13655060

[26] McInnes IB Schett G The pathogenesis of rheumatoid arthritis. N Engl J Med 2011;365:2205–19. 22150039 10.1056/NEJMra1004965

[27] Cooles FA Isaacs JD Pathophysiology of rheumatoid arthritis. Curr Opin Rheumatol 2011;23:233–40. 21427580 10.1097/BOR.0b013e32834518a3

[28] Kurko J Besenyei T Laki J Genetics of rheumatoid arthritis: a comprehensive review. Clin Rev Allergy Immunol 2013;45:170–9. 23288628 10.1007/s12016-012-8346-7 PMC3655138

[29] Viatte S Plant D Raychaudhuri S Genetics and epigenetics of rheumatoid arthritis. Nat Rev Rheumatol 2013;9:141–53. 23381558 10.1038/nrrheum.2012.237 PMC3694322

[30] Lee YH Bae SC Kim JH Meta-analysis of genetic polymorphisms in programmed cell death 1. Associations with rheumatoid arthritis, ankylosing spondylitis, and type 1 diabetes susceptibility. Z Rheumatol 2015;74:230–9. 24942602 10.1007/s00393-014-1415-y

[31] Yang M Zou Y Bai Y The programmed cell death 1 gene polymorphisms (PD 1.3 G/A, PD 1.5 C/T and PD 1.9 C/T) and susceptibility to ankylosing spondylitis: a meta-analysis. J Orthop Sci 2015;20:55–63. 25270018 10.1007/s00776-014-0648-6

[32] de Vooght KM van Wijk R van Solinge WW Management of gene promoter mutations in molecular diagnostics. Clin Chem 2009;55:698–708. 19246615 10.1373/clinchem.2008.120931

